# Comparing CAT12 and VBM8 for Detecting Brain Morphological Abnormalities in Temporal Lobe Epilepsy

**DOI:** 10.3389/fneur.2017.00428

**Published:** 2017-08-24

**Authors:** Farnaz Farokhian, Iman Beheshti, Daichi Sone, Hiroshi Matsuda

**Affiliations:** ^1^Integrative Brain Imaging Center, National Center of Neurology and Psychiatry, Tokyo, Japan; ^2^College of Life Science and Bioengineering, Beijing University of Technology, Beijing, China

**Keywords:** voxel-based morphometry, VBM8, CAT12, temporal lobe epilepsy, hippocampus sclerosis, statistical parameter mapping

## Abstract

The identification of the brain morphological alterations that play important roles in neurodegenerative/neurological diseases will contribute to our understanding of the causes of these diseases. Various automated software programs are designed to provide an automatic framework to detect brain morphological changes in structural magnetic resonance imaging (MRI) data. A voxel-based morphometry (VBM) analysis can also be used for the detection of brain volumetric abnormalities. Here, we compared gray matter (GM) and white matter (WM) abnormality results obtained by a VBM analysis using the Computational Anatomy Toolbox (CAT12) via the current version of Statistical Parametric Mapping software (SPM12) with the results obtained by a VBM analysis using the VBM8 toolbox implemented in the older software SPM8, in adult temporal lobe epilepsy (TLE) patients with (*n* = 51) and without (*n* = 57) hippocampus sclerosis (HS), compared to healthy adult controls (*n* = 28). The VBM analysis using CAT12 showed that compared to the healthy controls, significant GM and WM reductions were located in ipsilateral mesial temporal lobes in the TLE-HS patients, and slight GM amygdala swelling was present in the right TLE-no patients (*n* = 27). In contrast, the VBM analysis via the VBM8 toolbox showed significant GM and WM reductions only in the left TLE-HS patients (*n* = 25) compared to the healthy controls. Our findings thus demonstrate that compared to VBM8, a VBM analysis using CAT12 provides a more accurate volumetric analysis of the brain regions in TLE. Our results further indicate that a VBM analysis using CAT12 is more robust and accurate against volumetric alterations than the VBM8 toolbox.

## Introduction

Identifying brain morphological changes is a challenging task in neuroimaging studies. Voxel-based morphometry (VBM), introduced by Ashburner and Friston (1), is an advanced and powerful quantitative magnetic resonance imaging (MRI) procedure used to detect the brain morphological/volumetric changes in brain diseases. VBM assesses whole-brain structures with voxel-by-voxel comparisons, and it was developed to analyze tissue concentrations or volumes between subject groups in order to distinguish the structural abnormalities in the brain. The use of VBM contributes to investigations of the local alterations in tissue volume with high regional specificity throughout the brain (2).

The brain volumetric changes in Alzheimer’s disease (3), Parkinson disease (4), epilepsy (5), and the aging process (6) have been subjected to VBM analyses. Briefly, a standard VBM analysis incorporates the following preprocessing steps:

1. Tissue segmentation: the aim of this step is to classify the MRI scans into white matter (WM), gray matter (GM), and cerebrospinal fluid (CSF) images.
2. Spatial normalization: this step contributes to the alignment of the images by registering the MRI images to a standard Montreal Neurological Institute (MNI) space^1^ for the global brain shape, and by correcting the differences in the subjects’ head positions or orientation during scanning.
  A VBM analysis generally uses two registration methods: affine registration and non-linear registration. The affine registration is a linear mapping method that is used to achieve a global geometric transformation of the brain images, and this method is applied identically to each part of the image. With the non-linear registration, a finer-resolution match between images is achieved by allowing local transformations that adjust the different parts of each image in different manners.
3. Modulating: this step contributes to the correction of changes in the volume of the segmented images by applying a linear deformation or a non-linear deformation.
4. Smoothing: in the smoothing step, the segmented images are convolved with the use of an isotropic Gaussian kernel. This step helps to increase the signal-to-noise ratio, reducing the impact of misregistration between images and benefits on the normality of the statistics. Gaussian kernel sizes between 8 and 14 mm are usually used.
5. Matrix design: here, a structural measures model and a general linear model (GLM) are used to test hypotheses regarding the brain structures. Modeling brain imaging data using a GLM is described in greater detail in Ref. (7).
6. Statistical inference: a statistical inference analysis is conducted to identify any significant differences between subject groups.

The details of a standard VBM procedure have been described (2). The results of a VBM analysis are strongly dependent on the abovementioned steps and their respective algorithms, and these are especially critical for some brain diseases in which there are only small volumetric alterations in the patients compared to healthy subjects. The Structural Brain Mapping Group at the University of Jena (Jena, Germany)^2^ designed automatic and easy-to-use toolboxes named the VBM toolbox and the Computational Anatomy Toolbox (CAT) for performing comprehensive VBM analyses of brain structures. These toolboxes are implemented in Statistical Parametric Mapping (SPM) software (8).

**Figure 1:**
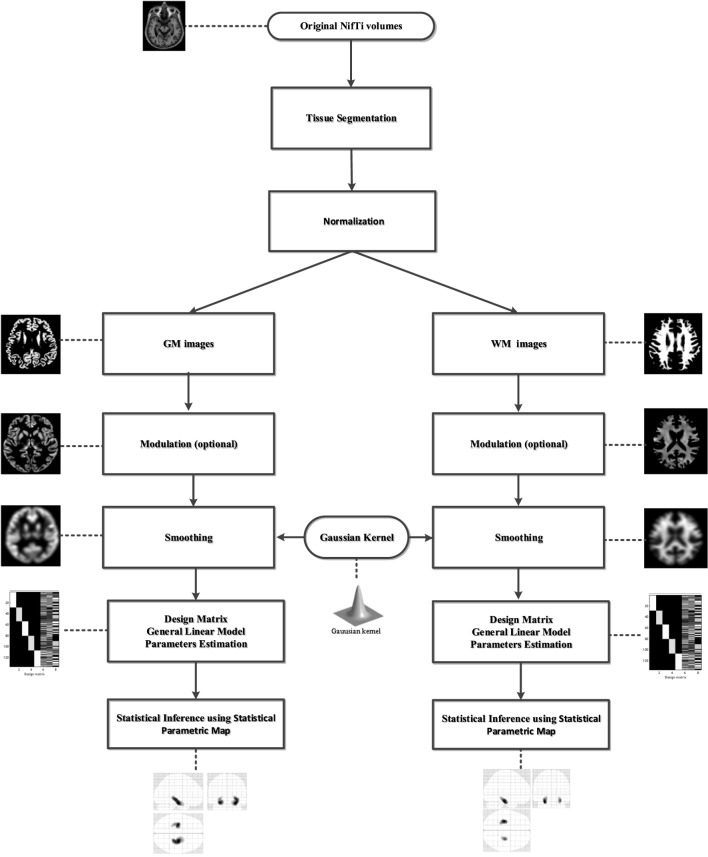
The processing framework in a standard voxel-based morphometry analysis using Statistical Parametric Mapping software. GM, gray matter; WM, white matter.

The VBM8^3^ toolbox runs within SPM version 8,^4^ and the CAT12 toolbox^5^ runs within SPM12 software.^6^ Figure 1 illustrates the processing steps of a standard VBM analysis performed to identify significant GM and WM alterations with the use of SPM software.

In this study, we compared the results obtained using the CAT12 toolbox and those using the older software program VBM8 in whole-brain VBM analyses conducted to identify significant brain morphological abnormalities in five groups of adult subjects: (1) healthy controls (*n* = 28), (2) right temporal lobe epilepsy (TLE) patients with hippocampus sclerosis (HS) (RTLE-HS; *n* = 26), (3) right TLE patients without HS (RTLE-no; *n* = 30), (4) left TLE patients with HS (LTLE-HS; *n* = 25), and (5) left TLE patients without HS (RTLE-no; *n* = 27). The CAT12 and VBM8 toolboxes are both currently widely used to perform VBM analyses in various brain diseases (9–11).

## Experimental Procedures

### Data Collection

All data used in this study were obtained from the National Center of Neurology and Psychiatry Hospital (Tokyo) for patients examined during the period from November 2013 through January 2017. The MRI scans were acquired from 3 T scanners manufactured by Philips (Best, The Netherlands) with the Digital Imaging and Communications in Medicine (DICOM) format with following protocol: repetition time/echo time: 7.12 ms/3.4 ms; flip angle: 10°; number of excitations: 1; 0.81 mm × 0.81 mm in plane resolution, 0.6-mm effective slice thickness with no gap, 300 slices, matrix of 260 × cm 320 cm; 26 cm × 24 cm field of view; acquisition time 4:01 min.

Table 1 summarizes the details of the demographic and clinical characteristics of the patients and healthy controls. There was no significant difference in age among the five groups (*F*-test = 0.91, *p* = 0.45). The TLE diagnosis was based on clinical symptoms and electroencephalography findings such as the presence of simple or complex partial seizures consistent with TLE, and focal epileptiform discharge predominantly in a unilateral temporal area as observed on a conventional scalp electroencephalogram.

**Table 1:** Characteristics of the healthy controls and TLE patients.

	HC (*n* = 28)	RTLE-HS (*n* = 26)	RTLE-no (*n* = 30)	LTLE-HS (*n* = 25)	RTLE-no (*n* = 27)
Age, years (mean ± SD)	40.67 ± 10.97	42.07 ± 11.53	43.76 ± 13.78	38.00 ± 13.11	39.14 ± 13.12
Female/male	12/16	14/12	14/16	17/8	14/13

*HC, healthy control; TLE, temporal lobe epilepsy; HS, hippocampus sclerosis; R, right; L, left*.

The patients with an HS or non-HS diagnosis were assessed by visual inspections of MRI findings, and thus the patients with an HS diagnosis were recognized based on different criteria: ipsilateral reduced hippocampal volume; increased T2 signal on the hippocampus; and abnormal morphology (i.e., a loss of internal architecture of the stratum radiatum, a thin layer of WM that separates the dentate nucleus and Ammon’s horn). All participants gave written informed consent for their data to be used in this study and to be published. The study was approved by the Institutional Review Board at the National Center of Neurology and Psychiatry Hospital.

### Methods and Statistical Analysis

As the first step, we reviewed and converted the raw DICOM scans into the Neuroimaging Informatics Technology Initiative format, using MRICRON software.^7^ To compare the findings revealed by the VBM analyses conducted with SPM12 and SPM8, we performed the preprocessing steps using the VBM8 and CAT12 toolboxes with the default setting, respectively. Briefly, in both the VBM8 and CAT12 toolboxes, all 3D T1-weighted MRI scans are normalized using a affine followed by non-linear registration, corrected for bias field in homogeneities, and then segmented into GM, WM, and CSF components (12). For both procedures, we used the Diffeomorphic Anatomic Registration Through Exponentiated Lie algebra algorithm (DARTEL) to normalize the segmented scans into a standard MNI space (13). Compared to the conventional algorithm, the DARTEL approach can provide more precise spatial normalization to the template (3, 14, 15). The details of a comparison between the DARTEL approach and the standard registration methods have been described (16).

In the present study, as part of the modulation step we performed a non-linear deformation on the normalized segmented images with both the VBM8 and CAT12 toolboxes. This modulation provides a comparison of the absolute amounts of tissue corrected for individual differences in brain size (17).

To identify the GM and WM morphological abnormalities in the present study’s TLE patients with and without HS, we used the GM and WM images. All segmented, modulated, and normalized GM and WM images were smoothed using 8-mm full-width-half-maximum Gaussian smoothing and then fed into a flexible factorial analysis in SPM8 and SPM12, separately.

In both the VBM8 and CAT12 toolboxes, the total GM volume, WM volume, and CSF volume were obtained, separately, on the basis of segmented images. The total intracranial volume (TIV) was calculated as the sum of the GM, WM, and CSF volumes for each toolbox, separately. As some authors have described using the age, gender, and head size of subjects in MRI studies (18), we used the subject’s age, gender, and respective TIVs in the present study’ matrix design. It should be noted that the same design is used for both the VBM8 and CAT12 toolboxes. The GM and WM morphological abnormalities are reported after using a family-wise error (FWE) with a *p*-value <0.05. The extent threshold was set at 100 voxels.

We conducted an analysis of variance followed by Tukey’s multiple comparison test for the statistical analysis of the demographics among the five groups. We accepted probability values (*p*) <0.05 as significant. All of the statistical analyses were done using SPSS software, ver. 16.0 (IBM-SPSS, Armonk, NY, USA).

## Results

### The VBM Analyses of the GM

**Figure 2:**
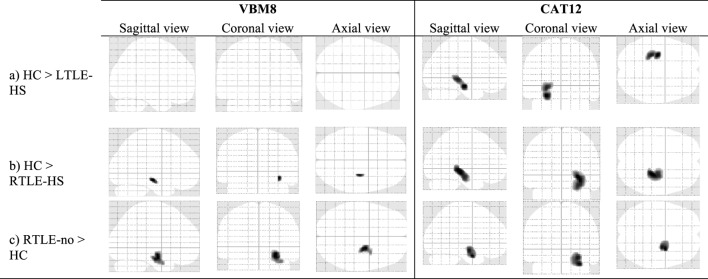
The significant alterations of regional gray matter (GM) volume revealed by the voxel-based morphometry (VBM) analyses using VBM8 versus CAT12. Family-wise error corrected at *p* < 0.05 and extend threshold *K* = 100.

Figure 2 and Table 2 show the significant GM volume alterations revealed by the two VBM analyses in the five subject groups using the VBM8 and CAT12 toolboxes; the VBM analysis conducted using CAT12 revealed a significant reduction in the GM volume at left and right hippocampus regions in the LTLE-HS and RTLE-HS subjects, respectively, compared to the healthy controls. In contrast, the VBM analysis results obtained with the VBM8 toolbox showed only a slight reduction in GM volume at the right hippocampus region in the RTLE-HS patients compared to the healthy controls.

**Table 2:** Clusters of GM alterations shown by the VBM analysis using VBM8 versus CAT12.

	Analysis	Location of peak voxels	Hemisphere	Cluster size (no of voxels)	Talairach coordinates (*x*, *y*, *z*)	MNI coordinates (*x*, *y*, *z*)	*T*-value (peak voxel)
VBM8	(a) HC > LTLE-HS	–	–	–	–	–	–
	(b) HC > RTLE-HS	Hippocampus	R	110	31 −21 −8	33 −19 −14	6.30
	(c) RTLE-no > HC	Amygdala	R	935	25 −11 −10	26 −9 −17	7.21
CAT12	(a) HC > LTLE-HS	Hippocampus	L	1,281	−26 −18 −11	−27 −16 −18	7.93
	(b) HC > RTLE-HS	Hippocampus	R	2,201	27 −28 −2	28 −27 −8	10.36
	(c) RTLE-no > HC	Amygdala	R	1,256	29 −5 −19	30 −2 −27	4.8

*Anatomical regions were derived from the Talairach Client program*.

*L, heft hemisphere; R, right hemisphere; MNI, Montreal Neurological Institute (FWE-corrected at *p* < 0.05); GM, gray matter; HC, healthy control; VBM, voxel-based morphometry; FEW, family-wise error*.

The VBM analyses using the VBM8 and CAT12 procedures each revealed a significant increase in the GM in the right amygdala in the RTLE-no patients compared to the healthy controls. Both the VBM8 and CAT12 procedures showed no significant GM volume alterations in the LTLE-no patients compared to the healthy controls or significant differences in the reverse contrast between these groups.

### The VBM Analyses of the WM

**Figure 3:**
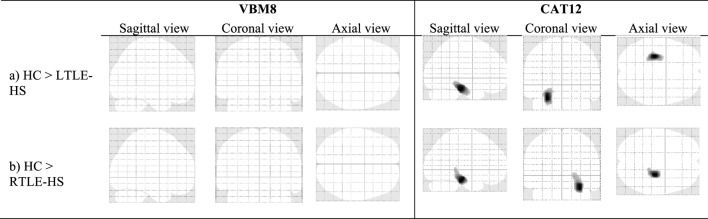
The significant alterations of regional white matter (WM) volume shown by voxel-based morphometry (VBM) analyses using VBM8 and CAT12. family-wise error (FWE) corrected at *p* < 0.05 and extend threshold *K* = 100.

The significant WM volume alterations in the five subject groups revealed by the VBM analyses using the VBM8 and CAT12 toolboxes are shown in Figure 3 and Table 3; the VBM analysis using the CAT12 toolbox identified a significant reduction in WM at the left and right parahippocampal regions in the LTLE-HS and RTLE-HS patients, respectively, whereas the VBM analysis using the VBM8 toolbox did not identify this abnormality in the LTLE-HS and RTLE-no patients. For both VBM analyses, there were no significant WM volume alterations in the LTLE-no and RTLE-no groups compared with the healthy controls, or in the reverse contrast.

**Table 3:** Clusters of WM alterations shown by the VBM analysis using VBM8 versus CAT12.

	Analysis	Location of peak voxels	Hemisphere	Cluster size (no of voxels)	Talairach coordinates (*x*, *y*, *z*)	MNI coordinates (*x*, *y*, *z*)	*T*-value (peak voxel)
VBM8	(a) HC > LTLE-HS	–	–	–	–	–	–
	(b) HC > RTLE-HS	–	–	–	–	–	–
CAT12	(a) HC > LTLE-HS	Para hippocampal	L	1,376	−26 −28 −17	−28 −27 −24	8.10
	(b) HC > RTLE-HS	Para hippocampal	R	1,291	27 −23 −14	28 −22 −21	7.98

*Anatomical regions were derived from the Talairach Client program*.

*L, heft hemisphere; R, right hemisphere; MNI, Montreal Neurological Institute (FWE-corrected at *p* < 0.05); FWE, family-wise error; HC, healthy control; VBM, voxel-based morphometry; WM, white matter*.

## Discussion

The reliability of different automatic brain segmentation programs such as SPM, FreeSurfer, and FSL was recently evaluated in patients with Alzheimer’s disease or mild cognitive impairment (19), and when MRIs with limited image quality were examined, the segmentation results obtained using the SPM program were more robust than those obtained using FreeSurfer or FSL (19). Based on those findings, we decided to use two versions of the widely applied SPM toolbox (i.e., VBM8 and CAT12) in our study. We investigated the differences and overlaps between the GM and WM alteration findings in healthy controls and RTE-HS, RTLE-no, LTLE-HS, and LTLE-no patients revealed by VBM analyses conducted with these toolboxes. Our findings indicated different patterns of gray- and white-matter abnormalities in TLE based on the VBM8 and CAT12 programs, as we discuss in detail below.

### GM Alterations

In the VBM analysis using the older toolbox (i.e., VBM8), we observed a slight reduction in GM compared to the healthy controls only in the right hippocampus region of the RTLE-HS patients, whereas the VBM analysis using the newer program CAT12 revealed significant GM reductions at the left and right hippocampus regions in the LTLE-HS and RTLE-HS patients, respectively. Our VBM analysis with CAT12 results are in line with those of studies that reported ipsilateral mesial temporal volume reductions in the GM of TLE-HS patients compared to healthy individuals (20–22). In addition, the results we obtained using CAT12 are broadly consistent with the pathology-based knowledge describing neuronal loss in the hippocampus of TLE-HS patients (23). VBM results obtained using the CAT12 toolbox should, therefore, be considered more representative of GM atrophy in TLE.

In our direct comparisons between the patients with a non-HS diagnosis versus the healthy controls, we observed a significant amygdala GM swelling in the RTLE-no patients in the VBM analysis using the VBM8 toolbox and in the same analysis using the CAT12 toolbox. This finding is in agreement with those of earlier studies that demonstrated TLE with amygdala enlargement (24–27).

### WM Alterations

Our comparison of WM alterations in our TLE-HS patients versus the healthy controls showed that ipsilateral mesial temporal WM reductions were identified by the VBM analysis using CAT12, whereas the VBM analysis using VBM8 did not detect any WM reduction in the LTLE-HS and RTLE-HS patients. The reason for this may be due to the improved and/or new segmentation algorithms incorporated into SPM12 compared to SPM8. Our VBM analysis with CAT12 findings are broadly consistent with studies describing ipsilateral WM abnormalities in TLE-HS patients compared to healthy controls (21, 28). VBM results obtained using the CAT12 toolbox should thus be considered more representative of WM atrophy in TLE compared to VBM results obtained with VBM8.

One limitation of our study might be that the subject groups were gender imbalanced; the LTLE-HS group in particular was predominantly female, and the healthy controls were mostly male. In addition, given that statistical significance can sometimes be affected by various factors, we should pay careful attentions to interpreting the significance of the results.

The authors in Ref. (29) compared the amygdala and hippocampus volumes using FreeSurfer and VBM8 procedures with manual segmentation. As part of a future study, we plan to evaluate the amygdala and hippocampus volumes as the main regions affected by epilepsy, using different approaches such as SPM (i.e., VBM8, CAT12), FreeSurfer, and FSL with manual segmentation in TLE patients. Although in the present investigation we used robust statistics and obtained results that are concordant with past studies, further studies using different samples and methods could be informative.

## Conclusion

To identify the brain morphological changes in TLE patients with and without HS, we performed two whole-brain VBM analyses—one using the toolbox VBM8 and the other using the CAT12 toolbox. These analyses provided disparate results. The results of the two analyses demonstrated that compared to the use of VBM8, a VBM analysis using the CAT12 toolbox identifies brain morphological abnormalities in patients with TLE that are more consistent with the literature- and pathology-based knowledge of TLE. The reason for this may be various improvements of the normalization and segmentation methods provided by SPM12 compared to the older program SPM8. It should be noted that the DARTEL process of normalizing to an averaged group template is not updated in SPM12 (30).

Our findings also demonstrate that brain morphological abnormalities in TLE patients identified using CAT12 are consistent with other studies that investigated the gray- and white-matter abnormalities in TLE using different methods such as optimized VBM (21) and diffusion tensor imaging (28). Thus, a VBM analysis using the CAT12 toolbox can contribute to a better detection of volumetric alterations compared to the use of VBM8. We suggest that future VBM analyses use the CAT12 toolbox as an advanced neuroimaging procedure in regional volumetric studies.

## Author Contributions

In this work, FF, IB, DS and HM contributed equally.

## Conflict of Interest Statement

The authors declare that the research was conducted in the absence of any commercial or financial relationships that could be construed as a potential conflict of interest.

## Footnotes

^1^http://www.mni.mcgill.ca/.

^2^http://www.neuro.uni-jena.de/.

^3^http://www.neuro.uni-jena.de/vbm/.

^4^http://www.fil.ion.ucl.ac.uk/spm/software/spm8/.

^5^http://www.neuro.uni-jena.de/cat/.

^6^http://www.fil.ion.ucl.ac.uk/spm/software/spm12/.

^7^www.mccauslandcenter.sc.edu/crnl/mricron/.
